# Cardioprotection against Heart Failure by Shenfu Injection via TGF-*β*/Smads Signaling Pathway

**DOI:** 10.1155/2017/7083016

**Published:** 2017-06-15

**Authors:** Jingyu Ni, Yang Shi, Lan Li, Jingrui Chen, Lingyan Li, Min Li, Jinqiang Zhu, Yan Zhu, Guanwei Fan

**Affiliations:** ^1^First Teaching Hospital of Tianjin University of Traditional Chinese Medicine, Tianjin 300193, China; ^2^Tianjin State Key Laboratory of Modern Chinese Medicine, Tianjin 300193, China; ^3^Ministry of Education, Key Laboratory of Formula of Traditional Chinese Medicine, Tianjin 300193, China; ^4^Tianjin Key Laboratory of Traditional Chinese Medicine Pharmacology, Tianjin University of Traditional Chinese Medicine, Tianjin 300193, China

## Abstract

**Objective:**

To explore the potential cardioprotective mechanism of Shenfu injection (SFI) against heart failure (HF) by attenuating myocardial fibrosis and cardiac remodeling.

**Methods and Results:**

Four weeks after myocardial infarction (MI), adult male Sprague Dawley rats were randomized for 4-week treatment with Valsartan, SFI, or vehicle. Echocardiography and hemodynamics were applied to evaluate cardiac functions. Myocardia of coronary artery ligated (CAD) rats were observed to investigate changes in cardiac structure and function. Our findings suggest that treatment with SFI could inhibit progression of myocardial fibrosis and attenuate cardiac remodeling. In addition, SFI decreased expression of Smad2 and Smad3, while increasing the expression of Smad7 through regulation of TGF-*β*/Smads signaling pathway.

**Conclusion:**

Treatment with SFI in Sprague Dawley rats improves ventricular structure and function and reduces cardiac fibrosis by ameliorating TGF-*β*/Smads signaling pathway after ventricular remodeling.

## 1. Introduction

Congestive heart failure (CHF) consists of a series of clinical syndromes characterized by systolic or diastolic dysfunction. CHF may result from a variety of pathogenic factors that result in reduced cardiac pumping ability. This subsequently leads to failure of the heart to meet the circulatory demands for physiological metabolism [1, 2]. CHF could lead to various clinical symptoms including dyspnea, fatigue, and fluid retention. Available data from an epidemiological study enrolling 15,518 adults aged 35–74 years suggest that the incidence of heart failure in China is about 0.9%, with 0.7% and 1.0% as the ratio of male to female patients respectively [3]. However, in western countries, CHF remains one of the top three leading causes of death in spite of contemporary therapeutic interventions. Thus, CHF with the high morbidity and mortality rates remains a public healthcare concern [4, 5].

Myocardial fibrosis (MF) is caused by various pathological factors which lead to the pathogenesis of heart disease till apposite phase time and is also the main cause of ventricular remodeling [6, 7]. Contemporary studies reveal a close relationship between myocardial fibrosis and various cardiovascular diseases, such as ischemic cardiomyopathy, hypertrophic cardiomyopathy, hypertensive heart disease, dilated cardiomyopathy, viral myocarditis, and diabetic cardiomyopathy. In brief, MF is a frequent outcome of the majority of heart diseases that eventually lead to CHF as previously described [8].

MicroRNAs (miRNAs) are a class of over 5000 noncoding RNAs that regulate more than half of the protein-encoding genes by provoking their degradation or preventing their translation [9]. Therefore, miRNAs are essential regulators of complex biological processes underlying several cardiovascular disorders, including left ventricular hypertrophy, ischemic heart disease, heart failure, hypertension, and arrhythmias. Moreover, circulating miRNAs serve as biomarkers in acute myocardial infarction and heart failure. In the past several years, microRNAs (miRNAs) have been shown to play critical roles in antiheart failure through cytoplasm and nucleotide binding to influence signaling pathways [10].

Transforming growth factor-beta (TGF-*β*) is a cellular factor that contains numerous members and is synthesized by a multiplicity of cells. TGF-*β* involves a number of essential physiological activities such as embryonic development, immune regulation, and angiogenesis. Human TGF-*β* superfamily contains at least 33 members, including TGF-*β* isoforms, activins, bone morphogenetic protein, and growth differentiation factor [11]. However, TGF-*β*1 is a cytokine which is closely related to tissue fibrosis in the TGF-*β* isoforms and has been implicated in the development of myocardial fibrosis [12]. In addition, TGF-*β*1 exerts its biological effect through TGF-*β*/Smads signaling pathway including the following 3 steps: First, TGF-*β* binds to receptors on the cell surface to constitute a heterotrimer. Second, the heterotrimer generates signal transduction in the cytoplasm by activating R-Smad protein. Finally, the transduction is transferred to the cell nucleus, binding to the target gene and regulating protein synthesis after a combination of R-Smad and Co-Smad [13–15].

Shenfu injection (SFI) has been used in disease management since 1987. The SFI, which originated from “Ji Sheng Fang,” has been used for nearly 30 years in China and exerted therapeutic effects. The active components of SFI are extracted from Radix Ginseng and Radix Aconiti Carmichaeli, which exert effects on increasing coronary blood flow to protect myocardium [16]. Recently, its use in the management of CHF, ischemia-reperfusion injury, arrhythmia, shock, myocardial infarction, coronary heart disease, and other cardiovascular diseases has been reported [17–21]. The present study was designed to identify TGF-*β*/Smads signaling pathway as a target for SFI to attenuate myocardial fibrosis and cardiac remodeling during CHF.

## 2. Materials and Methods

### 2.1. Animals

All animal experiments were performed in accordance with the guidelines of Tianjin University of TCM Animal Research Committee. Male Sprague Dawley (SD) rats, 6–8-week-old weighing 230~250 g, were purchased from Beijing Vital River Laboratories Co., Ltd., Beijing, China (certificate number SCXK [Jing] 2012-0001). The SD rats were raised in Tianjin University of TCM animal center at a temperature of 24 ± 2°C and humidity 40%  ± 5% and received standard diet and water. Before the experiment began, the rats were fasted for 12 hours but were permitted free access to water.

### 2.2. Drug and Reagents

SFI (batch number: 141001010) was obtained from Ya'an 999 Pharmaceutical Group Co. Ltd. (Ya'an, Sichuan Province, China), which was extracted from Radix Ginseng rubra and monkshood, prepared water soluble constituents into injection. Its manufacturing process was in compliance with the guidelines of Good Manufacturing Practice and Good Laboratory Practice, and the major components of SFI were confirmed by the Chinese government agency. Valsartan (batch number: X1711) was purchased from Novartis Pharmaceutical Co. Ltd. (Beijing, China) and prepared to aqueous solution with ultra-pure water. Penicillin sodium for injection (batch number: 017140617) was purchased from Shijiazhuang Pharmaceutical Co. Ltd. (Shijiazhuang, Hebei Province, China), prepared to 0.4 million U per milliliter solution with physiological saline before experiment.

Chloral hydrate (batch number: Q/12HB 4218-2010) was purchased from Tianjin Kermel Chemical Reagent Co., Ltd. (Tianjin, China), dissolved with physiological saline at 5% concentration before experiment. The N-terminal probrain natriuretic peptide (NT-proBNP) assay kit, matrix metalloproteinase-9 (MMP-9) assay kit, connective tissue growth factor (CTGF) assay kit, and transforming growth factor-beta 1 (TGF-*β*1) assay kit were purchased from Uscn Life Science Inc. (Wuhan, Hubei Province, China). Hematoxylin-eosin (HE) staining reagent was purchased from Nanjing Senbeijia biological technology Co., Ltd. (Nanjing, Jiangsu Province, China). Masson trichrome staining reagent was obtained from Sigma-Aldrich Co., LLC. (St. Louis, MO, USA).

### 2.3. HF Model and Drug Administration

The SD rats were randomly divided into five groups. For HF model preparation, rats were anesthetized with 6 mL/kg chloral hydrate by peritoneal injection and placed in a supine position. Surgery of left thoracotomy was performed under stereomicroscope at the surgical site, in order to observe the heart clearly. After the heart was exposed adequately, the proximal left anterior descending coronary artery was ligated with a 6/0 silk. Then the chest was closed to allow spontaneous respiration and recovery. The rats were released and laid on an electric heating blanket. The same procedure was performed in the rats belonging to the sham group but without ligation of suture silk [19]. All animals were reared in cages for 4 weeks after surgery.

After 4 weeks, the rats except the sham group were screened to determine left ventricular ejection fraction at 30%~35% by echocardiographic detection and defined as HF model. The rats in treatment groups were given Valsartan aqueous solution or SFI via gavage or intramuscular injection at the dose of 10 mg/kg or 1.5 mL/kg and 0.75 mL/kg for 4 weeks, respectively. Besides, animals in the sham group were given physiological saline through peritoneal injection at 10 mL/kg. The care and administrative procedure in this current study were in accordance with the clinical guidelines [22].

### 2.4. Echocardiographic Detection of Left Ventricular Function

A Vevo2100 High Resolution Ultrasound System in real time (VisualSonics Vevo 2100, Canada) with an MS-250 ultrasound scanning transducer (model C5) was used to measure left ventricular function of rats [23]. We used mask with isoflurane at a concentration of 1.5% to anesthetize animals. Moreover, the chest was shaved and placed in the supine position on a 37° platform. We squeezed ultrasound coupling agent from bottle and spread it evenly on the chest of the animal using cotton swabs. M-mode and Doppler-mode images were recorded from the long axis of the left ventricles. Inherent measurement package of ultrasound system was applied to measure the following parameters about function and structure of left ventricles: left ventricle ejection fraction percentage (EF%), left ventricle fractional shortening percentage (FS%), left ventricular internal diameter diastole (LVIDd), left ventricular posterior wall diastole (LVPWd), left ventricular internal diameter systole (LVIDs), and aortic flow peak velocity (AV Peak V) [24]. Each data was measured three times and the mean value was calculated.

### 2.5. Hemodynamic Evaluation of Cardiac Function

Four weeks after drug administration, the cannulation which was connected with PowerLab 8 passages electrophysiolograph (ADInstruments, PowerLab 8/30, New South Wales, Australia) was prepared to access from right carotid artery to the left ventricle after the rats were anesthetized with 5% chloral hydrate (6 mL/kg) by peritoneal injection. The following parameters were measured as indicators of hemodynamics: heart rate (HR), left ventricular pressure (LVP), left ventricular systolic pressure (LVSP), left ventricular end diastolic pressure (LVEDP), left ventricular maximum upstroke velocity (+d*p*/d*t*_max_), and left ventricular maximum descent velocity (−d*p*/d*t*_min_). All data were analyzed off-line with LabChart7 at the end of the study on the personal computer [25].

### 2.6. miRNA Expression Profiling

MiRNAs from rat heart were isolated with the mirVana miRNA Isolation Kit (AM1556, Applied Biosystems, Foster City, USA). Affymetrix miRNA V4.0 gene chip technology was used to analyze myocardial samples of heart failure rats. In this experiment, we compared every experimental groups each other, screening 29 miRNAs on common differential expression. Picking out 7 miRNAs associated with heart failure and taking statistical analysis.

### 2.7. Quantitative Real-Time PCR

Total RNA was extracted with TRIzol reagent. Total RNA concentrations were measured using ND-1000 (NanoDrop Technologies), and cDNA was synthesized with high-capacity cDNA reverse transcription kits (Applied Biosystems, F. Hoffmann-La Roche Ltd., Switzerland), according to the protocol provided by the manufacturer. PCR primers for TGF-*β*1, Smad2, Smad3, Smad7, and GAPDH were designed and synthesized by Sangon Biotech Co., Ltd. (Shanghai, China). The sequences of the forward and reverse primers used for amplification are shown in Table 1. Real-time PCR was performed using a one-step real-time PCR system (Applied Biosystems) with SYBR green fluorophore. Each sample for reactions was run in at least duplicates. Threshold cycle (Ct) data were collected by an inherent system and posted on an Excel sheet. GAPDH was performed as an internal control. 2^−ΔΔCt^ was calculated to evaluate mRNA fold change relative to GAPDH [26].

### 2.8. Detection of Serum Biochemical Indicators

Rats were positioned in a supine position after anesthetized with 5% chloral hydrate (6 mL/kg) by peritoneal injection. The abdomen was split to isolate the abdominal aorta. Blood was collected from the abdominal aorta by syringes then centrifuged at 1500 rpm for 10 minutes. The content of NT-proBNP, TGF-*β*1, MMP-9, and CTGF of serum was detected with an enzyme linked immunosorbent assay (ELISA) kit according to the manufacturer's instructions.

### 2.9. Histological Assessment of Cardiac Tissue

Animals were euthanized in states of being anesthetized. The heart was then removed from the thorax, infiltrated in 50 mL centrifugal tubes filled with 4% paraformaldehyde solution for more than 48 hours. Paraffin-embedded left ventricular tissue sections (4 *μ*m) were stained with hematoxylin-eosin and Masson trichrome staining reagent, respectively. Light microscope and micropolariscope were implemented to analyze the sections. The images were captured using a camera connected to an optical microscope (Digital Sight Lecica DM3000, Leica Microsystems Wetzlar, Germany) and processed with the Leica Application Suite (Leica Application Suite, Leica Microsystems Wetzlar, Germany).

### 2.10. Statistical Analysis

All data were expressed as means ± SD. SPSS 17.0 statistical software was applied to treat statistical analysis. One-way ANOVA and Tukey test for multiple comparisons were used to identify statistical significance. A value of *P* < 0.05 was regarded as statistically significant.

## 3. Results

### 3.1. Echocardiography

**Figure 1:**
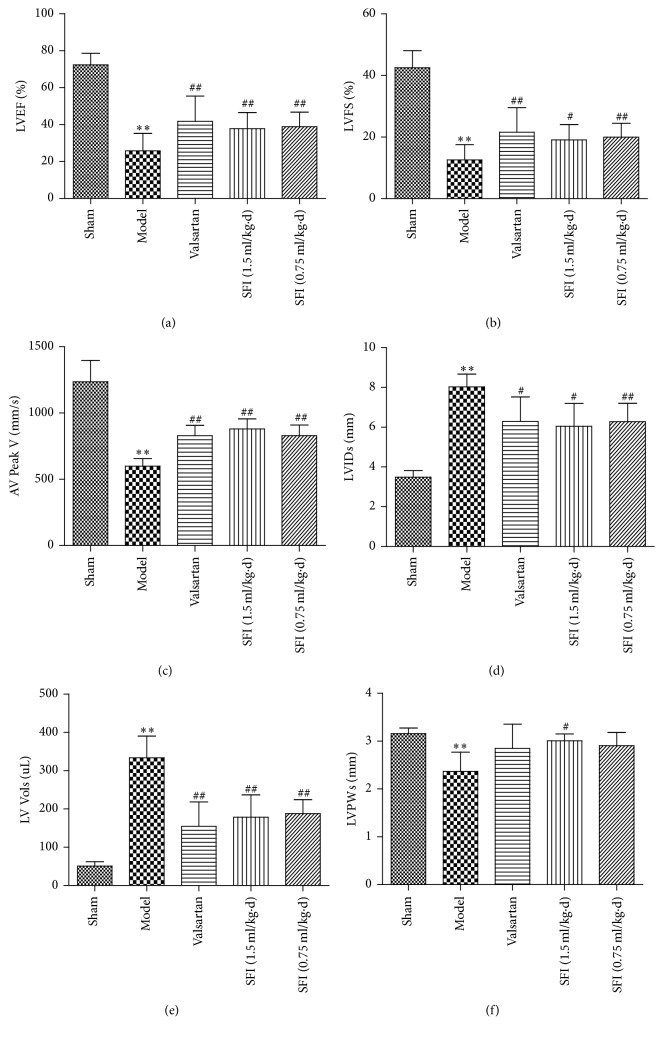
Echocardiographic characterization of cardiac systolic function in rats subjected to HF.* Notes*. Quantitative assessment of dilation and systolic function based on LVEF (a), LVFS (b), AV Peak V (c), LVIDs (d), LV systolic volume (LV Vols) (e), and LVPWs (f). Data are expressed as mean ± SD from ten animals. ^*∗∗*^*P* < 0.01 compared with the sham group; ^#^*P* < 0.05 and ^##^*P* < 0.01 compared with the HF group.

**Figure 2:**
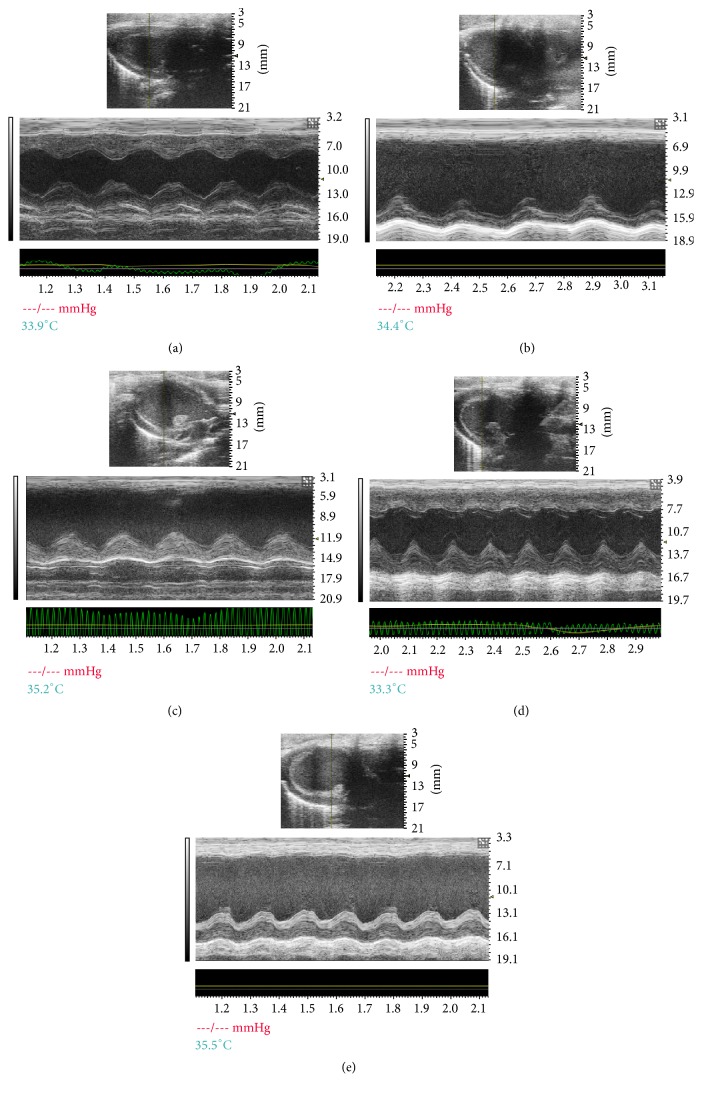
Representative echocardiographic images (M-mode) in different groups.* Notes*. (a) Sham group, (b) HF group, (c) Valsartan + HF group, (d) SFI 1.5 mL/kg + HF group, and (e) SFI 0.75 mL/kg + HF group. HF, heart failure; SFI, Shenfu injection.

The benefit of SFI treatment on cardiac damage triggered by HF was estimated through quantitative analysis of echocardiograms. The results of echocardiography assessment for various groups are shown in Figure 1, and the HF group emerged significantly increased in LVIDs and left ventricular systolic volume (LV Vols). The decreases in EF%, FS% and AV Peak V for the HF group were improved remarkably 4 weeks of SFI treatments. Meanwhile, SFI at a dose of 1.5 mL/kg enhanced LVPWs in value of *P* < 0.05. The typical echocardiograms on M-mode of different groups are demonstrated in Figure 2.

**Figure 3:**
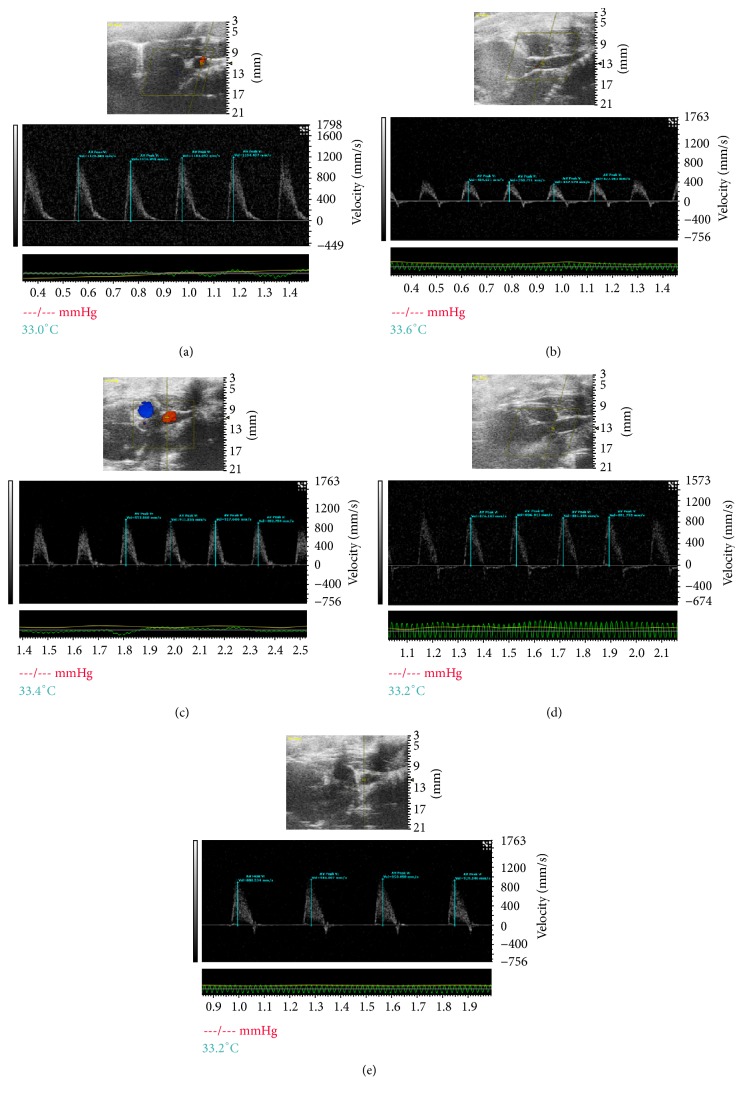
Representative transmitral valvular flow profiles and the color images acquired by color Doppler ultrasonography after 4-week drug administration, where the white shadow represents the aortic flow velocity.* Notes*. (a) Sham group, (b) HF group, (c) Valsartan + HF group, (d) SFI 1.5 mL/kg + HF group, and (e) SFI 0.75 mL/kg + HF group. HF, heart failure; SFI, Shenfu injection.

As shown in Figure 3, color Doppler ultrasonography was used to acquire the images after 4 weeks of drug administration. The white shadow on images of Figure 3 represents the aortic flow velocity. As it was presented, the HF group had markedly lower maximum blood flow velocity and velocity time integral than that in the sham group. On the contrary, we observed significantly higher maximum blood flow velocity and velocity time integral after SFI treatment (1.5 mL/kg and 0.75 mL/kg). The result was quantified and shown in Figure 1(c).

### 3.2. Hemodynamic Analysis

**Figure 4:**
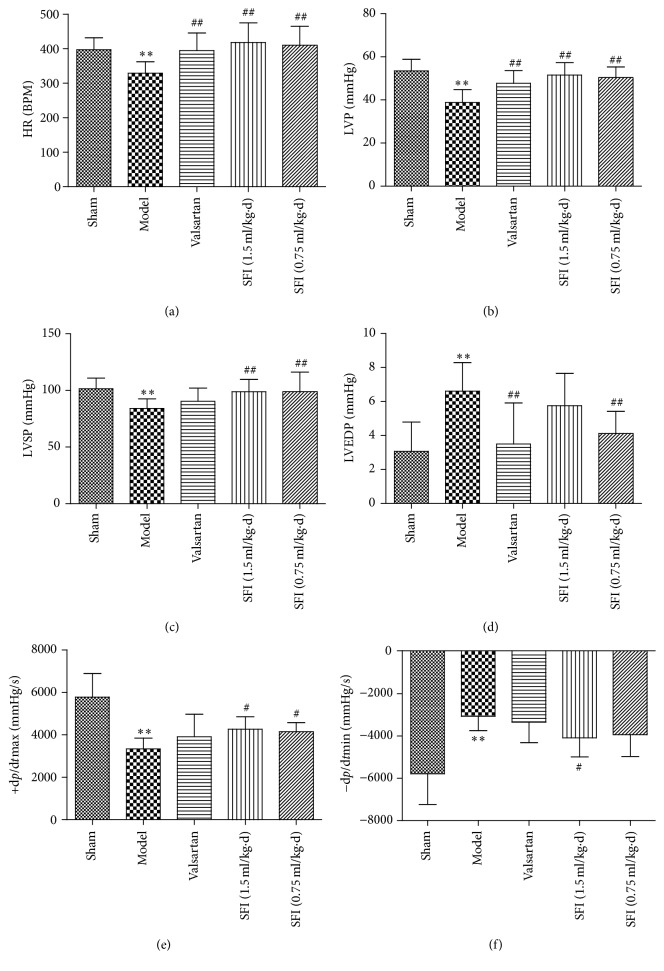
Hemodynamic assessment was performed to the left ventricle through right carotid artery to evaluate the role of SFI treatment in the management of HF.* Notes*. Quantitative assessment of hemodynamic on cardiac function based on HR (a), LVP (b), LVSP (c), LVEDP (d), +d*p*/d*t*_max_ (e), and −d*p*/d*t*_min_ (f). Data are expressed as mean ± SD from eight animals. ^*∗∗*^*P* < 0.01 compared with the sham group; ^#^*P* < 0.05 and ^##^*P* < 0.01 compared with the HF group.

Hemodynamics of cardiac organ for 5 groups were detected in order to confirm whether HF could benefit from SFI. As shown in Figure 4, compared with the sham group, HF caused a conspicuous decline in heart rate, left ventricular pressure, and left ventricular systolic pressure. In addition, left ventricular development pressure, +d*p*/d*t*_max_ and −d*p*/d*t*_min_, was decreased substantially in HF group, indicating a severe damage to heart function. However, these impairments were improved by treatment with SFI.

### 3.3. Different Expression miRNA of Three Groups

**Figure 5:**
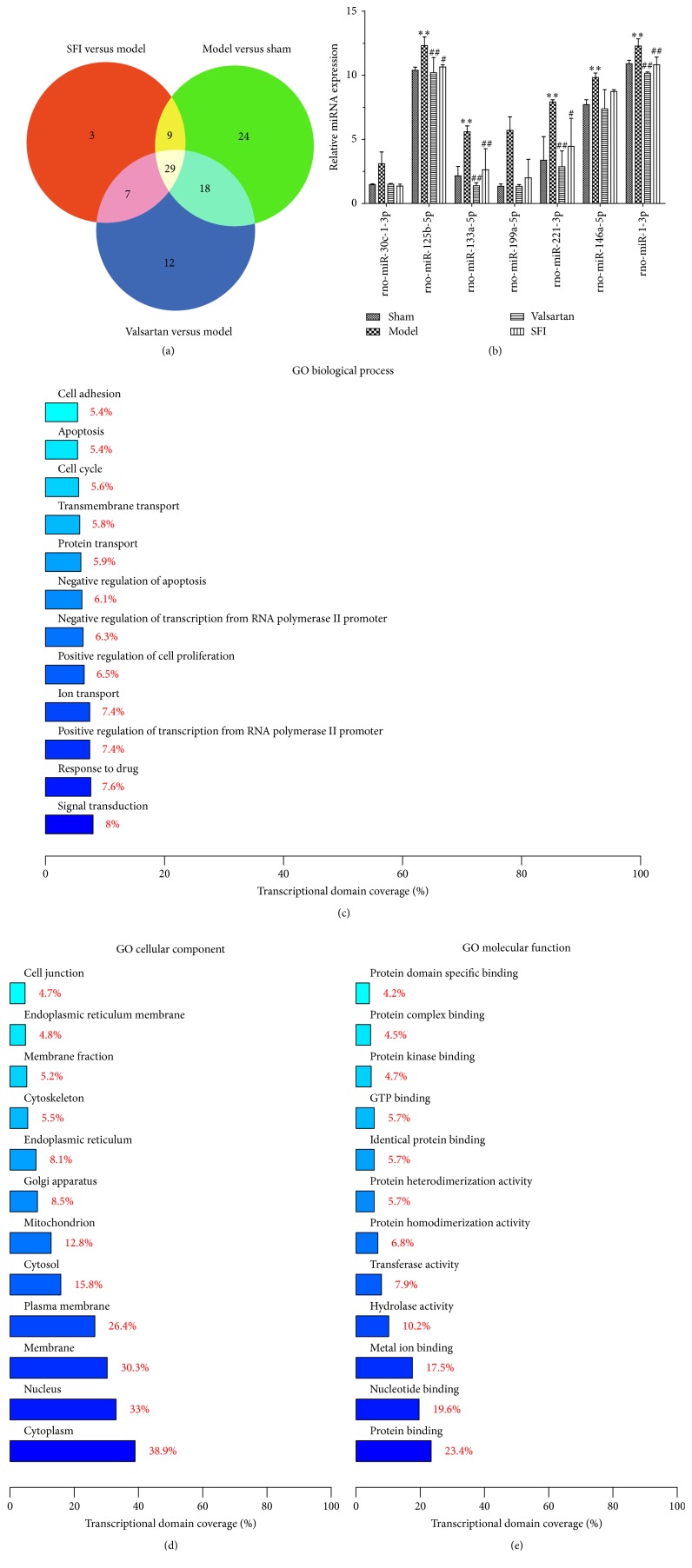
The experimental study of expression for Shenfu injection on myocardial miRNAs in rats with chronic congestive heart failure. (a) The intersection diagram of differentially expressed miRNA in three experiment groups. (b) Statistical analysis of seven miRNA expression differences in heart failure (*n* = 3). (c–e) GO analysis of differential genes.* Notes*. Compared with the sham group, ^*∗∗*^*P* < 0.01; compared with model group, ^#^*P* < 0.05 and ^##^*P* < 0.01.

In this experiment, we compared every experimental group with each other, screening 29 miRNAs on common differential expression (Figure 5(a)). Picking out 7 miRNAs associated with heart failure and taking statistical analysis, we found that Shenfu injection could significantly downregulate the levels of rno-miR-30c-1-3p, rno-miR-125b-5p, rno-miR-133a-5p, rno-miR-199a-5p, rno-miR-221-3p, rno-miR-146a-5p, and rno-miR-1-3p (Figure 5(b)). Meanwhile, there was statistical difference among them (Table 2, *P* < 0.05 or *P* < 0.01). GO analysis shows that the different genes were linked to signal transduction, cytoplasm, and nucleotide binding (Figures 5(c)–5(e)). Overall, Shenfu injection participated in downregulation of miRNAs associated with CHF progress. Thus, Shenfu injection could play the role of antiheart failure therapy through cytoplasm and nucleotide binding to influence signaling transduction pathways.

### 3.4. Effects of SFI on Serum NT-proBNP, TGF-*β*1, MMP-9, and CTGF

**Figure 6:**
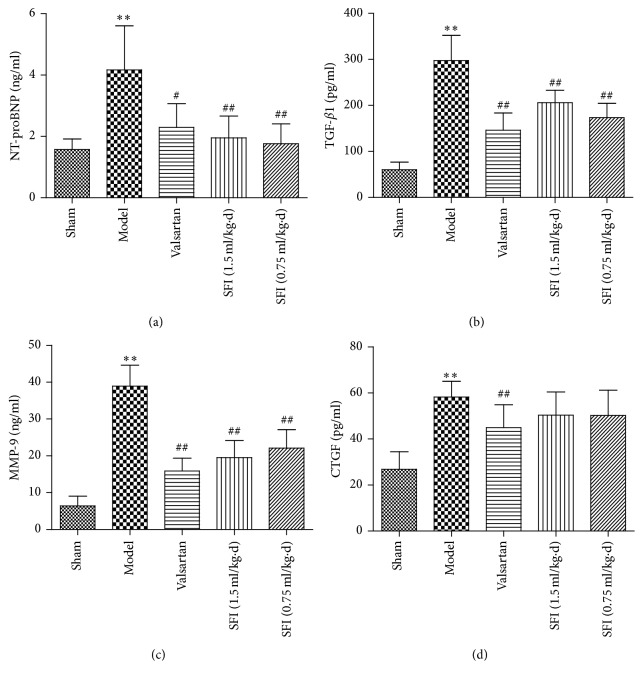
The effect of SFI on the content of NT-proBNP, TGF-*β*1, MMP-9, and CTGF of rat serum.* Notes*. Statistical results from ELISA for NT-proBNP (a), TGF-*β*1 (b), MMP-9 (c), and CTGF (d). Data are expressed as mean ± SD from ten animals. ^*∗∗*^*P* < 0.01 compared with the sham group; ^#^*P* < 0.05 and ^##^*P* < 0.01 compared with the HF group. NT-proBNP, N-terminal probrain natriuretic peptide; TGF-*β*1, transforming growth factor-beta 1; MMP-9, matrix metalloproteinase-9; CTGF, connective tissue growth factor; SD, standard deviation; HF, heart failure; SFI, Shenfu injection.

To investigate the situation of heart function and myocardial fibrosis, NT-proBNP, TGF-*β*1, MMP-9, and CTGF levels were determined by ELISA. In comparison to the sham group, the model group expressed higher levels of NT-proBNP, TGF-*β*1, MMP-9, and CTGF in statistical difference. However, both SFI 1.5 mL/kg and SFI 0.75 mL/kg recorded a potential result in improving heart function and attenuating myocardial fibrosis (Figure 6). Unfortunately, SFI with the two doses studied possessed little significant effect on the CTGF level.

### 3.5. Pathological Analysis of Cardiac Tissue

#### 3.5.1. Hematoxylin-Eosin (HE) Staining

**Figure 7:**
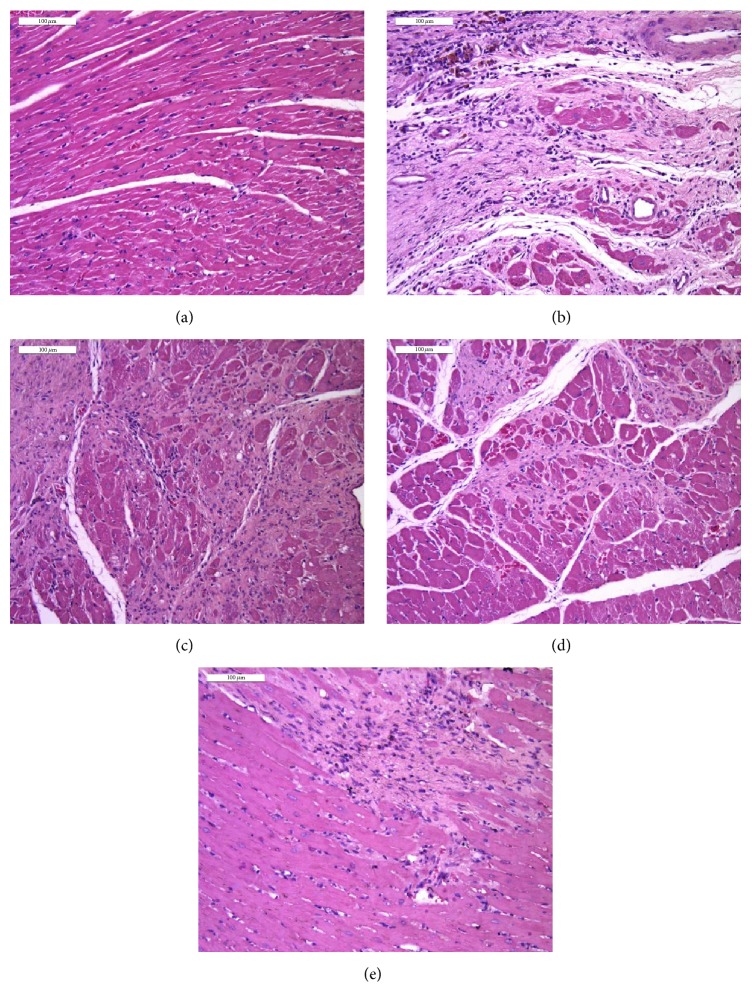
The effect of SFI treatment on myocardial pathology in rats with HE staining after 4 weeks.* Notes*. (a) Sham group, (b) HF model group, (c) Valsartan + HF group, (d) SFI 1.5 mL/kg + HF group, and (e) SFI 0.75 mL/kg + HF group. Bar, 100 *μ*m. HF, heart failure; SFI, Shenfu injection; HE, hematoxylin-eosin.

To further evaluate whether SFI plays a paramount role in HF-induced structural modification and cardiac remodeling, we examined left ventricle myocardial tissues of rats with pathological section staining. The results of HE staining are shown in Figure 7. Compared with the sham group (a), infarct areas were observed in the HF group (b) where myocardial tissues altered significantly, including myocardial interstitial edema, infiltration of leukocytes, and rupture of myocardial fibers. These lesions were attenuated by 4-week treatment with SFI (c, SFI 1.5 mL/kg + HF group; d, SFI 0.75 mL/kg + HF group).

#### 3.5.2. Masson Trichrome Staining

**Figure 8:**
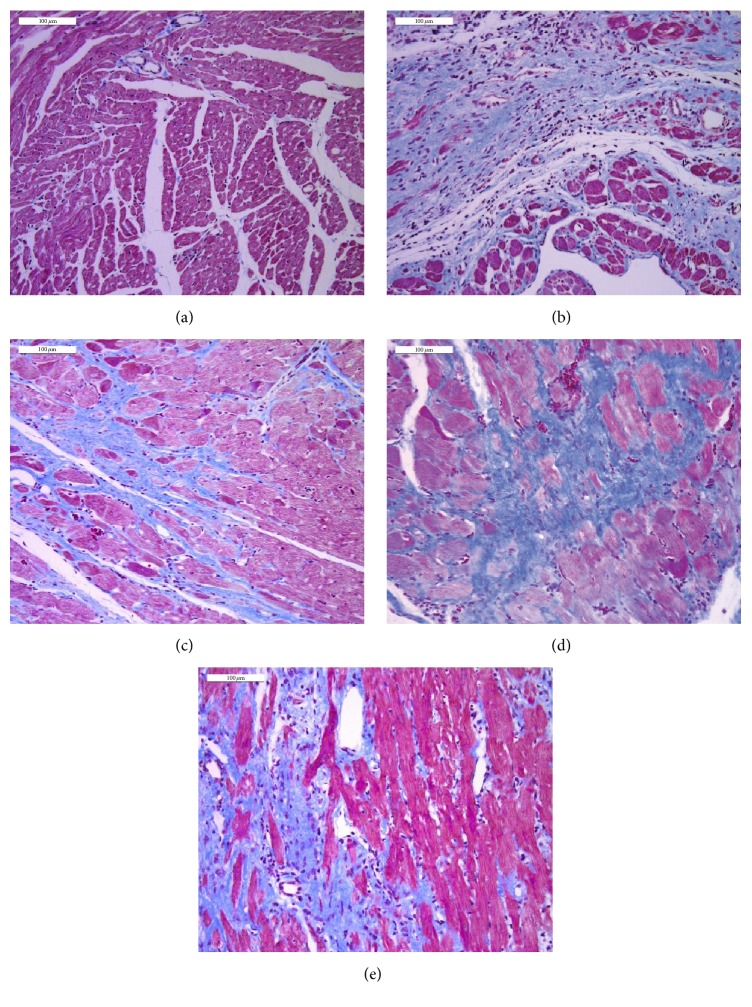
The effect of SFI treatment on left ventricular myocardial fibrosis in rats with Masson trichrome staining after 4 weeks.* Notes*. (a) Sham group, (b) HF model group, (c) Valsartan + HF group, (d) SFI 1.5 mL/kg + HF group, and (e) SFI 0.75 mL/kg + HF group. Myocardium was stained red and collagens were stained blue. Bar, 100 *μ*m. HF, heart failure; SFI, Shenfu injection.

To explore myocardial fibrosis and cardiac remodeling in left ventricle, we performed Masson trichrome staining to confirm our supposition. The results of Masson trichrome staining are presented in Figure 8. Compared with the sham group (a), remarkable collagen accumulation occurred in the border area of infarction of the myocardial tissues of the HF group (b), indicating that left ventricle remodeled severely. Fortunately, this condition was ameliorated through inhibiting myocardial fibrosis by SFI (c, SFI 1.5 mL/kg + HF group; d, SFI 0.75 mL/kg + HF group).

### 3.6. Study on TGF-*β*/Smads Signaling Pathway through RT-PCR

**Figure 9:**
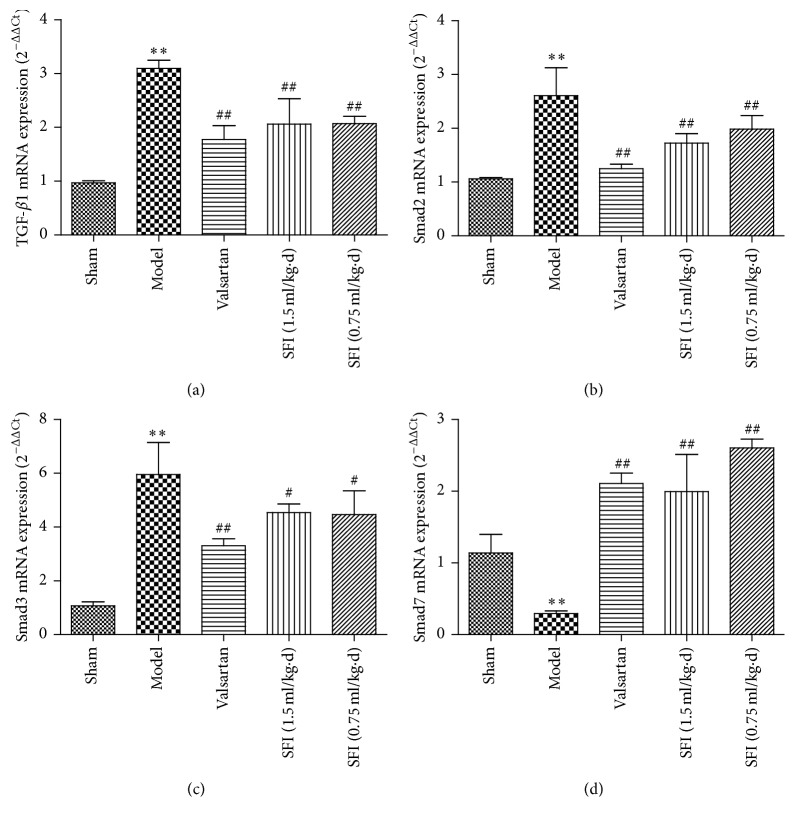
SFI regulates expression of TGF-*β*1, Smad2, Smad3, and Smad7 mRNA in rat hearts.* Notes*. The relative levels of cardiac TGF-*β*1 (a), Smad2 (b), Smad3 (c), and Smad7 (d) mRNA were assessed by real-time PCR. Results were normalized to GAPDH. Data are expressed as mean ± SD (each group, *n* = 3). ^*∗∗*^*P* < 0.01 compared with the sham group; ^#^*P* < 0.05 and ^##^*P* < 0.01 compared with the HF model group. SFI, Shenfu injection; HF, heart failure; SD, standard deviation; PCR, polymerase chain reaction; GAPDH, glyceraldehyde-3-phosphate dehydrogenase; TGF-*β*1, transforming growth factor-beta 1.

TGF-*β*/Smads signaling pathway is reported as a principal mechanism of myocardial fibrosis and cardiac remodeling. Therefore, study on SFI activity against HF via TGF-*β*/Smads signaling pathway is imperative. Reverse transcription polymerase chain reaction (RT-PCR) experiment was performed to quantify TGF-*β*1, Smad2, Smad3, and Smad7 mRNA at the end of SFI treatment (Figure 9). The TGF-*β*1, Smad2, and Smad3 mRNA levels (Figures 9(a)to 9(c)) were significantly increased in the HF group (*P* < 0.01) as compared to the sham group, while those in SFI groups were dramatically lower than the HF group (*P* < 0.05, *P* < 0.01). However, Smad7 mRNA level was in opposite trend with the above-mentioned three mRNAs. Briefly, there was an observed significant decrease in Smad7 mRNA expression in the HF group, but the levels were elevated in SFI groups (Figure 9(d)). In conclusion, treatment with SFI targets TGF-*β*/Smads signaling pathway by upregulating the levels of TGF-*β*1, Smad2, and Smad3 while downregulating Smad7 level.

## 4. Discussion

In China, TCM plays an integral role in the clinical management of ailments. SFI which is based on TCM theory has been widely used in treating cardiovascular diseases especially, CHF [20]. Since SFI is approved by China Food and Drugs Board (CFDA) and applied in many hospitals and clinics, it has obtained significant curative effects from patients and medical practitioners [27]. In this study, we found that treatment with SFI through intramuscular injection could protect myocardium against MF via TGF-*β*/Smads signaling pathway during CHF process.

The data obtained from ultrasonic imaging and the studied hemodynamics parameters including the enhancement of LVEF, FS, AV Peak V, LVP, and LVSP suggest that SFI could improve cardiac function in a rat model of CHF. These experimental results imply that SFI treatment could significantly ameliorate left ventricular contractility, increase coronary blood flow, and relieve symptoms of heart failure in a rat model of CHF.

MiRNAs are key regulators of complex biological processes underlying several cardiovascular disorders, including left ventricular remodeling and myocardial fibrosis. In our study, we screened 29 miRNAs on common differential expression by Affymetrix miRNA V4.0 microarray experiments. Picking out 7 miRNAs associated with heart failure and taking statistical analysis, our data reveal that Shenfu injection could significantly downregulate the levels of rno-miR-30c-1-3p, rno-miR-125b-5p, rno-miR-133a-5p, rno-miR-199a-5p, rno-miR-221-3p,rno-miR-146a-5p, and rno-miR-1-3p. There was statistical difference between the groups. Our analysis shows that SFI elicits antiheart failure by downregulating miRNAs that have been implicated in heart failure pathology. Among these antiheart failure-associated miRNAs, we explicitly focused on miR-146a, which was predicted to influence myocardial fibrosis through TGF-beta 1/Smad signal transduction pathway during CHF [28, 29]. At the same time, miR-125b and miR-199a were implicated in myocardial signaling networks triggering fibrosis [30]. And, TGF-*β*1 is a cytokine which is closely related to tissue fibrosis in the TGF-*β* isoforms and has been implicated in the development of myocardial fibrosis. Therefore, based on the miRNA expression profile, we focus on the TGF-*β*/Smads signaling pathway.

TGF-*β*/Smads is known to be a classical cell signaling pathway during disease progression. Recent studies suggest that TGF-*β*/Smads signaling pathway plays a crucial role in the pathogenesis of MF. Data from previous studies suggest that receptor regulated Smads; Smad2 and Smad3 are phosphorylated by activated transforming growth factor-*β* receptor I. The phosphorylated Smads then form a Smad compound through the combination with Smad4 (common-partner Smads). Moreover, the compound is transferred into the nucleus immediately and interacts with various transcription factors to regulate the gene transfer and exert biological effects. However, inhibitory Smads (Smad6 and Smad7) and receptor regulated Smads could interact through competitive binding to prevent phosphorylation of receptor regulated Smads thereby, blocking the action of TGF-*β* [13–15]. That is to say, this study detected four TGF-*β*/Smads signaling pathway genes, such as TGF-*β*1, Smad2, Smad3, and Smad7 by RT-PCR in order to confirm the effect of SFI for the signaling pathway and to further support our hypothesis. As aforementioned, it is apparent that expression of TGF-*β*1, Smad2, and Smad3 was substantially increased in myocardial tissue with CHF preconditioning. However, after treatment with SFI, levels of these three genes expression were markedly reduced. On the contrary, Smad7 expression level increased remarkably [31–33]. Thus, the cardioprotection of SFI treatment in CHF for myocardial fibrosis may be achieved by upregulation of Smad7 and downregulation of TGF-*β*1, Smad2, and Smad3 gene expression via TGF-*β*/Smads signaling pathway.

Ventricular remodeling is a series of cardiac dysfunction characterized by morphological changes in myocardial cells, collagen lattices, and blood vessel bed and is critical in CHF development [33]. Furthermore, ventricular remodeling has close relationship with MF, which is demonstrated by cell proliferation and excessive accumulation of extracellular matrix, while the most important regulatory cytokines remain TGF-*β*1, MMP-9, and CTGF. The present study found significant increased TGF-*β*1, MMP-9, and CTGF levels in serum of model rats. Conversely, we observed decreased levels of TGF-*β*1, MMP-9, and CTGF in the sham, Valsartan, and SFI administration group. This suggests that treatment with SFI could attenuate MF as well as left ventricular remodeling [34].

To further provide evidence to support our findings, we performed two additionally relative experiments including HE staining and Masson trichrome staining. Results obtained suggest that SFI could inhibit cardiac fibrosis and reduce collagen accumulation. Data from biochemical indicators in serum and histological sections support the evidence obtained through echocardiography and hemodynamics studies.

Valsartan, which is an angiotensin type II receptor antagonist, was used as a positive control drug in our present study since it is widely documented to improve ventricular remodeling and prevents blood vessel damage [35–37].

In conclusion, the current study has demonstrated that treatment with SFI could lead to cardioprotection against MF and heart failure by targeting TGF-*β*/Smads signaling pathway and down regulation of miRNAs. Our results suggest that SFI confers protection against CHF through multitarget and multisignaling pathways. Thus, we have provided evidence to suggest that the cardioprotection against heart failure by SFI is at least in part through the regulation of TGF-*β*/Smads signaling pathway. Therefore, we have set the stage for future research focus on a causal link between the observed downregulation of miRNAs and the changes in the expression of TGF-*β*1, Smad2, Smad3, and Smad7 mRNA, involved in cardiac fibrosis and remodeling.

## Figures and Tables

**Table 1:** Nucleotide sequences of primers used in real-time PCR.

Gene	Primers	Nucleotide sequences 5′-3′
*TGF*-*β1*	Forward	*5*′-*CGCAACAACGCAATCTATG*-*3*′
	Reverse	*5*′-*ACCAAGGTAACGCCAGGA*-*3*′
*Smad2*	Forward	*5*′-*ACTATACCCACTCCATTCCA*-*3*′
	Reverse	*5*′-*CACTATCACTTAGGCACTCG*-*3*′
*Smad3*	Forward	*5*′-*CCAGTGCTACCTCCAGTGTT*-*3*′
	Reverse	*5*′-*CTGGTGGTCGCTAGTTTCTC*-*3*′
*Smad7*	Forward	*5*′-*GGCTTTCAGATTCCCAACTTC*-*3*′
	Reverse	*5*′-*GGCTTTCAGATTCCCAACTTC*-*3*′
*GAPDH*	Forward	*5*′-*ATGATTCTACCCACGGCAAG*-*3*′
	Reverse	*5*′-*CTGGAAGATGGTGATGGGTT*-*3*′

**Table 2:** Summary of the differences in the expression of seven miRNA related to heart failure.

miRNA name	Model versus sham	Valsartan versus model	SFI versus model
rno-miR-30c-1-3p	+2.098	−2.063	−2.268
rno-miR-125b-5p	+1.185	−1.207	−1.156
rno-miR-133a-5p	+2.588	−4.034	−2.119
rno-miR-199a-5p	+4.231	−4.257	−2.851
rno-miR-221-3p	+2.343	−2.758	−1.775
rno-miR-146a-5p	+1.273	−1.330	−1.126
rno-miR-1-3p	+1.126	−1.208	−1.134

*Note*. *n* = 3, “+” means upregulation, “−” means downregulation, and number means differential multiple of miRNA.
